# Leveraging transcriptome-wide association studies identifies the relationship between upper respiratory flora and cell type-specific gene expression in severe respiratory disease

**DOI:** 10.1371/journal.pone.0322864

**Published:** 2025-05-09

**Authors:** Lei Xu, Ran He, Xiangyu Ye, Yifan Wang, Shirong Hui, Haochang Li, Hongbo Chen, Peng Huang

**Affiliations:** 1 Depatment of Epidemiology, Center for Global Health, School of Public Health, National Vaccine Innovation Platiorm, Nanjing Medical University, Nanjing, China; 2 Department of Infectious Disease, Jurong Hospital Affiliated to Jiangsu University, Jurong, Jiangsu, China; West Bengal University of Animal and Fishery Sciences, INDIA

## Abstract

**Objectives**The upper respiratory tract flora may influence host immunity and modulate susceptibility to viral respiratory infections. This study aimed to investigate the associations between upper respiratory tract flora and immune cells in severe ILI, identify specific microbial taxa and immune response pathways contributing to disease severity, and elucidate how flora influences ILI progression by modulating immune cell functions.**Methods**Heritability of GWAS summary data was estimated using LDSC (v1.0.1). Gene-level genetic associations were analyzed with MAGMA. scRNA-seq data were integrated with genetic association data using scDRS. FUSION was used to construct cell type-specific expression quantitative trait locus models based on genotypes and scRNA-seq data from the onek1k project, which were combined with flora abundance-related GWAS data for a transcriptome-wide association study.**Results**From the LDSC analysis, data from 1195 severe ILI-associated GWASs with upper respiratory flora(h2 > 0.1) were included in subsequent analysis. TWAS identified 19 significant association pairs (*P*_*adj *_< 0.05), and 1226 differentially expressed genes between mild and severe ILI patients (*P*_*adj*_ < 0.05 and | log_2_FC|>0.25). Functional enrichment analyses using GO, KEGG, and Reactome databases revealed that immune cells,such as CD4 + T effector memory cells, cDCs, NK cells, were enriched in multiple biological processes or pathways.**Conclusions**This study identified associations between severe ILI-related upper respiratory tract flora and cell type-specific gene expression, potentially explaining how differential flora influences ILI progression. CD16 + monocytes exhibited the most differentially expressed genes, followed by proliferating cells and cDCs, highlighting the significant role of immune cell-enriched pathways in ILI progression.

## 1. Introduction

Influenza-like illness (ILI) is a global public health problem that kills hundreds of thousands of people each year [1–3]. Acute pulmonary inflammation and multiple organ failure associated with severe ILI are the leading causes of death after ILI infection [4–6]. Vaccination is the most effective way to prevent influenza. However, vaccine effectiveness has been low to moderate in recent years [7,8], and vaccine coverage remains low, particularly in low- and middle-income countries where pathogens are susceptible to mutation and protection is suboptimal [9]. Consequently, ILI infection remains a significant public health problem with enormous economic and social burdens globally. The study of ILI-organism interactions and mechanisms is important for infection control.

As more research supports the critical role of the flora in regulating host immunity [10–12], exploring whether these effects extend to influenza risk may provide new perspectives and approaches for refining prevention strategies. Animal models and human studies have shown that nasopharyngeal respiratory surfaces are colonized by large commensal flora [11–13]. They can alter susceptibility to viral respiratory infections, including influenza, by modulating host immunity. For example, flora can potentially inhibit the overgrowth of pathogens and prevent their transmission to the lungs, thereby serving as a “protective barrier” in maintaining respiratory health.[14] In recent years, it has been suggested that there is a relationship between the diversity of the upper respiratory tract flora and respiratory infections [15]. For example, one study reported that host susceptibility to ILI was closely related to the abundance of Streptococcus and Bacillus salivarius [16]. The abundances of Haemophilus, Prevotella, Clostridium and Neisseria in the oral cavity and nasopharynx were associated with the length of hospital stay in infants hospitalized with respiratory syncytial virus infection in one study [17]. A recent study of patients with novel coronavirus pneumonia (COVID-19) revealed significant differences in the diversity of the upper respiratory tract flora between COVID-19 patients and controls, with Corynebacterium, Lactobacillus, and other related flora consistently and significantly lower in COVID-19 patient samples, whereas Neisseria was consistently and significantly greater [18]. The epithelial cells of the upper and lower respiratory tracts are the primary target cells for influenza virus infection and replication. These epithelial cells are surrounded by a complex flora that may directly or indirectly interact with influenza viruses to mediate infection risk. Symbiotic strains can prevent infection by modulating the host’s innate and adaptive immune responses [10,11]. However, relatively few studies have been conducted on the relationship between the upper respiratory flora and the progression of ILI, and its mechanism in the progression of ILI has not been clarified. In-depth investigation and elucidation of the interactions and regulatory mechanisms between ILI and the host upper respiratory flora, lung injury, and systemic immune responses may provide new intervention strategies for ILI and help prevent and ameliorate infection-induced lung injury.

The antigen-specific immune response is an important means for the body to recognize and kill pathogens and has a significant impact on infection control and clinical resolution. Excessive immune killing can cause severe histopathological damage, leading to worsening of symptoms and even death [19–21]. In the intervening years, with the development of biotechnology, the understanding of immune cell function has deepened, and the role of different immune subtypes in the process of pathogen infection has been gradually recognized. For example, CD8 + effector memory T cells generated after atypical pneumonia can provide durable protection against coronaviruses. In addition, during respiratory syncytial virus infection, this subtype of T cells is associated with the development of pathological immune damage [22,23]. In COVID-19 patients, tissue-resident Th17 cells coordinate the killing effects of lung macrophages and cytotoxic CD8 + T cells and may be associated with a protective role against lung injury [24].

On the basis of the above findings, this study hypothesized that the upper respiratory tract flora may influence the course of ILI infection by modulating the recognition and killing effects of immune cells on pathogens after an organism is infected with ILI. To this end, We employed the method of Genome-Wide Association Study (GWAS), a well-established and efficient approach widely used to identify genetic loci associated with common diseases or traits [25]. The method aims to explore the relationship between specific traits or diseases and single nucleotide polymorphisms (SNPs), which represent variations in a single nucleotide within the genome. Typically, GWAS analyses rely on high-throughput genotyping technologies, such as SNP arrays, and incorporate statistical methods (e.g., logistic regression, mixed-effects models) to assess the association between each SNP and the trait of interest. By comparing genotype differences between individuals, GWAS can uncover genetic variations related to specific traits or disease risks. In recent years, the scale of GWAS has expanded to include over 10 million SNPs [26]. Since these SNPs are typically inherited in linkage disequilibrium blocks, certain representative or tag SNPs can capture the majority of variations within each block. Furthermore, with the increasing demand for explaining the genetic basis of more diseases or traits, as well as the substantial increase in sample sizes [27], the statistical power and reliability of the results have been significantly enhanced [28].Through Transcriptome-Wide Association Studies (TWAS), we integrated GWAS data with expression quantitative trait loci (eQTL) data [29]. TWAS not only focuses on the direct association between genotypes (such as SNPs) and traits, but also considers how gene expression levels in specific tissues or cell types influence the occurrence of traits or diseases. Although thousands of genomic loci related to complex traits have been identified through GWAS, most of the trait-associated variants are located in non-coding regions, the functionality of which remains difficult to interpret [30]. Moreover, identifying variants with small or moderate effects typically requires large sample sizes to achieve adequate statistical power. By aggregating multiple eQTLs to capture gene regulatory effects and directly measuring these effects at the transcriptome level, TWAS effectively overcomes these challenges, enabling the establishment of more biologically interpretable associations between genes and complex traits or diseases [31]. We first calculated the heritability of the upper respiratory tract flora data obtained. Second, PBMC samples from patients with severe influenza were categorized into two groups, ILI mild and ILI severe, on the basis of symptoms, to identify the types of upper respiratory tract flora associated with these conditions. Third, after performing quality control on the data from these two groups, we conducted horizontal genetic association analysis via MAGMA software to integrate the flora SNP signals at the gene level. The scDRS (single-cell disease relevance score) was subsequently used to calculate genetic signals associated with the flora to identify the relevant cell types. Fourth, cell type-specific eQTL models were constructed for each identified microbiome-associated cell type to identify specific genes. Finally, pathway enrichment analysis was conducted on the identified gene sets via R software.

## 2. Materials and methods

### 2.1 Collection of flora data

All adult Chinese individuals in this cohort were recruited for the multi-omics study, This cohort consists of 2,984 healthy individuals from the Chinese population. From 2017 to 2018, approximately 3,932 oral samples were newly collected from this cohort for whole metagenome sequencing, including 2,017 tongue dorsum samples and 1,915 saliva samples [32]. For the saliva sample, a double concentration of stabilizing reagent kit was used and 2ml saliva was collected. The samples from Yunnan Province were self-collected using a commercial kit (Catalog No. 401103, Zeesan, Xiamen, China) [33].

### 2.2 Data acquisition and processing for genome-wide association studies

(1) Data Acquisition for Genome-Wide Association Study of Upper Respiratory Tract Flora: GWAS data of upper respiratory tract flora were downloaded from the China National Gene Bank database (CNGBdb, project data No. CNP0001664) [32]. A total of 1,566 dorsal tongue flora and 1,547 salivary flora GWAS summary datasets were included in this project (genome version GRCh38). The full GWAS summary data were downloaded for this study. Notably, owing to some of the differences in the definitions of some of the floras between this study and the project, this study referred to the LPSN database (https://lpsn.dsmz.de/) for the GWAS summary data that had the same or most similar definitions to each of the respiratory tract flora [34].
(2) Data quality control: The following QC processes were also performed on the downloaded GWAS summary data related to upper respiratory tract flora: i) SNPs located on autosomes were retained; ii) SNPs detected in less than 70% of the samples were excluded; iii) SNPs with alleles other than “A”, “C”, “G”, or “T” were excluded; iv) SNPs with anomalies in the variance of the summary statistic (≤ 0); v) SNPs with abnormal standard error (SE ≤ 0) were excluded; and vi) SNPs with allele frequency (MAF) < 0.001.
(3) Heritability calculation: The heritability (h2) of the included GWAS summary data was estimated via LDSC software (v.1.0.1), with 504 East Asian (EAS) populations from the 1000 Genomes Project Phase III as the reference panel [35,36]. For GWAS data related to the upper respiratory tract flora, only those with heritability estimates (h2) greater than 0.1 were included in subsequent analyses.

### 2.3 Data acquisition and processing for single-cell sequencing

(1) Data acquisition for single-cell sequencing: In this study, two scRNA-seq datasets containing peripheral blood mononuclear cell (PBMC) samples from patients with severe ILI were acquired to identify cell types associated with the upper respiratory tract flora. The original study of the first scRNA-seq dataset (GSE149689) included a total of 20 PBMC samples from asymptomatic, mildly symptomatic, and severely symptomatic COVID-19 patients; patients with severe influenza; and healthy controls [37]. We selected 16 samples totaling 53,387 cells from patients hospitalized with COVID-19 and severe influenza for subsequent analysis. A second source study of scRNA-seq data (GSE164948) included 20 PBMC samples from patients hospitalized with COVID-19, influenza, and CAP infections with other pathogens, as well as healthy controls [38]. Similarly, 16 samples from hospitalized patients with a total of 13,999 cells were selected for subsequent analysis. Five samples from asymptomatic and mildly symptomatic COVID-19 patients were defined as having mild ILI, and the remaining 27 samples from patients with severe COVID-19, critically hospitalized patients and hospitalized patients with influenza or CAP were defined as having severe ILI.
(2)Data quality control:First, i) scDblFinder (v.1.9.4) was used to identify and remove potential doubles [39]; ii) cells expressing fewer than 200 genes, with a total UMI count ≥20,000, or expressing a percentage of mitochondrial genes ≥15% of the total UMI count were excluded; and iii) genes expressed in fewer than 3 cells were excluded. Finally, 51,243 cells and 23,442 genes were retained for subsequent analyses.
(3) Sample merging, dimensionality reduction and clustering: i) Seurat (v.4.4.0) was first employed to perform *SCTransform* normalization on each sample, in which a regression was performed on the percentage of mitochondrial gene expression in each cell [40,41]; ii) all samples were merged, 3,000 highly variable genes were selected, and linear dimensionality reduction was performed via principal component analysis; iii) batch correction of PCs was conducted on a per-sample basis via Harmony (v.1.2.0) [42]; iv) the top 28 Harmony-corrected PCs that cumulatively explained up to 90% of the variance were selected to construct a K-nearest neighbor graph structure and were analysed by clustering via Louvain’s algorithm; and v) uniform manifold approximation and projection (UMAP) with the same number of PCs for visualization.
(4) Cell type annotation: To ensure the accuracy of cell type annotation, we performed cell type annotation on the subpopulations obtained by clustering on the basis of widely used marker genes as well as those used in the two original studies of the data (Table 1). A total of 51,243 cells were finally divided into 15 subtypes. Of these, two subtypes, platelets (PLTs, n = 3,636) and red blood cells (RBCs, n = 829), were not the cell types.

**Table 1:** Cell types defined marker gene.

Cell Subtype	Cell Population	Marker Gene
T Cell	15715	CD2、CD3E
CD4 + naive T cell	5821	CD3E、CD4、CCR7、SELL
CD4 + Effector T Cell	360	CD4、GNLY、NKG7
CD4 + Effector Memory T Cell	716	CD4、CTLA4、GPR183
CD8 + Naive T Cell	760	CD3E、CD8A、CD8B、CCR7、SELL
CD8 + Effector T Cell	5022	CD8A、CD8B、GNLY、NKG7
CD8 + Effector Memory T Cell	3036	CD8A、CD8B、GZMK、GPR183
NKT	1382	CD3G、KLRF1、KLRD1
NK	6378	KLRF1、KLRD1
B Cell	6231	CD79A、MS4A1
Monocyte	16029	CD14、CD163、CD68
CD14 Mono	14377	CD14、S100A12
CD16 Mono	1652	CD14、FCGR3A
cDC	272	FCER1A、CD1C
Proliferating Cell	771	MKI67、TYMS
PLT	3636	PF4、PPBP
RBC	829	HBD、HBM

(5) Differential expression analysis: The differentially expressed genes (DEGs) between the severe and mild ILI groups in each cell type were analysed via the Wilcoxon rank sum test. Among them, genes whose |log_2_(fold change)|(|log_2_FC|) > 0.25 and BH-adjusted *P *< 0.05 were defined as differentially expressed genes.

### 2.4 Analysis of flora-associated cell types

The enrichment of flora-related genetic signals in 29,149 cells from the severe ILI samples was calculated via the single-cell disease relevance score (*scDRS,* v.1.0.3) to identify each flora-associated cell type [43]. In brief, given the processed scRNA-seq data and gene‒disease association results output from *MAGMA*, *scDRS* first constructs a putative disease gene set *G* with each g in *G* weighted by its GWAS *MAGMA* z score ($\omega_{g}$) and then quantifies the aggregate expression of the putative disease gene set in each cell to generate cell‒specific raw disease scores *S*_*c*_:


$$S_{c}=\frac{\sum_{g\in G}\omega_{g}\sigma_{tech,\;g}^{-1}X_{cg}}{\sum_{g\in G}\omega_{g}\sigma_{tech,\;g}^{-1}}$$

where $\sigma_{tech,\;g}$ indicates the estimated gene-specific technical noise of gene *g* and where $X_{cg}$ is the normalized expression of cell *c* on gene *g*. *scDRS* also uses Monte Carlo (MC) to sample matched control gene sets and generate a set of raw control scores. *scDRS* normalizes these raw scores and computes cell-level *P* values on the basis of the empirical distribution of the pooled normalized control scores for each cell *c*:


$$\begin{array}{c} P_{c}=\frac{1+\sum_{C^{\prime }=1}^{n_{cell}}\Sigma_{b=1}^{B}\Pi \left(S_{c}\leq S_{c^{\prime }b}^{ctrl}\right)}{1+n_{cell}B} \end{array}$$

where *B* is the number of MC samples. In this study, we draw the top 1,000 genes from *MAGMA* ranked by the z score to generate the disease gene set and set the MC sampling times to 1000.

### 2.5 Identification of flora-associated cell type-specific genes

For the identified cell types associated with each flora, the cell type-specific genes associated with each flora were identified by constructing a cell type-specific gene expression quantitative trait locus (eQTL) model and applying the analytical framework of FUSION software to perform TWAS analysis [29]. Only cis-eQTL relationships (cis-eQTLs) were considered in this study.

#### 2.5.1 Cell type-specific gene expression quantitative trait genome models.

(1) Genotyping Data Processing: The converted genotyping data were subjected to a series of QCs with reference to the original study [44], and genotypes were populated via minimac4 (v.4.1.6) software. After excluding SNPs with a fill score < 0.7 or an MAF < 0.01, a final set of 5,807,466 SNPs from 1034 individuals was obtained [35].
(2) Single-cell sequencing data processing: The QC steps applied to the scRNA-seq data included the following: i) exclusion of genes whose expression was detected in fewer than 3 cells; ii) exclusion of 18,959 cells from 15 subjects that were not included in the genotype resampling; and iii) considering the heterogeneity of populations, experimental conditions, and cell type definitions, a total of 883,855 cells from 19 subtypes were reclassified into 14 subtypes on the basis of the similarity of gene expression in the flora-associated cell type analysis section. (**Table 2**).

**Table 2:** onek1k Summary of single-cell data quality control.

onek1k Cell Subtype	Cell Count		Target Cell Subtype	Target Cell Count
	**Before Filtering**	**After Filtering**		
CD4 Naive	259012	255130	CD4 + Naive T Cell	255130
CD4 CTL	17993	17849	CD4 + Effector T Cell	17849
CD4 TEM	31261	30879	CD4 + Effector Memory T Cell	30879
CD8 Naive	52538	51487	CD8 + Naive T Cell	51487
CD8 TCM	16409	16175	CD8 + Effector T Cell	16175
CD8 TEM	161051	159634	CD8 + Effector Memory T Cell	159634
NK	163202	160465	NKT	160465
NK_CD56bright	7006	6922	NK	6922
B naive	65702	64321	B memory	123261
B intermediate	29889	29466	B memory	
B memory	30234	29474	B memory	
CD14 Mono	36130	35560	CD14 + Monocyte	35560
CD16 Mono	15743	15502	CD16 + Monocyte	15502
cDC1	114	114	cDC	4515
cDC2	4456	4401	cDC	
CD4 Proliferating	773	762	Proliferating Cell	2770
CD8 Proliferating	305	304	Proliferating Cell	
NK Proliferating	1731	1704	Proliferating Cell

(3) Model construction: Construct the cis-eQTL model via FUSION.compute_weights pipeline from FUSION [29]. The cis-eQTL model construction process consists of i) extracting SNPs located in the genetic cis-regulatory regions of the target genes (cis-SNPs) from the genotypic data of the study subjects via PLINK (v.1.9.0) software [45]; ii) a kinship matrix was constructed on the basis of the cis-SNPs of the target gene, and its heritability was calculated via GCTA (v.1.93.2 beta) in combination with the individual expression levels of the gene (cis-h2) [46]; iii) for genes with significant heritability, each cis-eQTL method was fitted in the form of cross-validation to estimate the effect of fitting the model via each method; and iv) each cis-eQTL method was fitted via all samples to obtain the final result of each cis-SNP gene expression weight.

#### 2.5.2 Identification of flora-associated cell type-specific genes.

On the basis of the constructed cell type-specific cis-eQTL model, genomic data from the EAS population at 1000 Genomes Project were used as a reference panel, and TWAS analysis was performed on each flora-associated GWAS summary dataset via the FUSION framework to identify the cell type-specific expressed genes associated with each flora. Considering that the eQTL model was constructed from the European(EUR)population and that the flora-associated GWAS summary data were from the EAS population, this part of the study focused only on nominally significant genes, i.e., those with uncorrected P < 0.05.

### 2.6 Functional enrichment analysis

For a given set of genes, pathway enrichment analysis was performed via the clusterProfiler (v.4.2.2) and ReactomePA (v.1.38.0) software packages on the basis of the Gene Ontology (GO), KEGG, and Reactome pathway databases [47,48].

## 3. Results

### 3.1 Analysis of GWAS data related to the flora of the upper respiratory tract and identification of associated cell types

A total of 1,566 GWAS summary statistics of the dorsal tongue flora and 1,547 GWAS summary statistics of the salivary flora were included in this part of the study. After QC, 5,345,095 SNPs were obtained. On the basis of the results of LDSC, 1195 GWASs of upper respiratory tract flora with h2 > 0.1 were included in the subsequent analyses, of which 489 were salivary flora-associated GWASs and 706 were dorsal tongue flora-associated GWASs. On the basis of MAGMA genetic association analysis, we identified 27 genes that were significantly associated with at least one flora. The top five genes, ranked in ascending order of P value, are *SLC2A9* (*P* = 1.37E-09), *SLC2A9* (*P* = 3.82E-08), *MDGA2* (*P* = 4.59E-08), *NUDT8* (*P* = 1.01E-07), and *BBOX1* (*P* = 1.40E-07) (Table 3 and An additional file shows this in more detail [see S1 Fig]). Notably, the *SLC2A9* gene was significantly associated with two flora in the GWAS, both of which belong to the genus Oribacterium within the phylum Firmicutes in the tongue dorsum microbiome.

**Table 3:** Genetic association analysis based on MAGMA gene level.

Gene	Chromosome	N_SNPs_	Ns_ample_	*P*	Sample	Bacterial community
*SLC2A9*	4	808	1996	1.37E-09	Tongue	k__Bacteria; p__Firmicutes; c__Clostridia; o__Lachnospirales; f__Lachnospiraceae; g__Oribacterium; s__unclassified_mgs_1215
*SLC2A9*	4	808	1991	3.82E-08	Tongue	k__Bacteria; p__Firmicutes; c__Clostridia; o__Lachnospirales; f__Lachnospiraceae; g__Oribacterium; s__unclassified_mgs_489
*MDGA2*	14	1636	2000	4.59E-08	Tongue	k__Bacteria; p__Firmicutes; c__Bacilli; o__Lactobacillales; f__Aerococcaceae; g__Granulicatella; s__unclassified_mgs_2134
*NUDT8*	11	64	1898	1.01E-07	Saliva	k__Bacteria; p__Firmicutes; c__Clostridia; o__TANB77; f__CAG-508; g__CAG-793; s__unclassified_mgs_3234
*BBOX1*	11	205	1985	1.40E-07	Tongue	k__Bacteria; p__Actinobacteriota; c__Actinobacteria; o__Actinomycetales; f__Actinomycetaceae; g__Pauljensenia; s__unclassified_mgs_3571
*NDUFV1*	11	50	1898	1.57E-07	Saliva	k__Bacteria; p__Firmicutes c__Clostridia; o__TANB77; f__CAG-508; g__CAG-79; | s__unclassified_mgs_3234
*GJB4*	1	78	1998	3.08E-07	Tongue	k__Bacteria; p__Spirochaetota; c__Spirochaetia; o__Treponematales; f__Treponemataceae; g__Treponema_D; s__unclassified_mgs_1124
*SPDYE18*	7	838	1900	3.20E-07	Saliva	k__Bacteria; p__Firmicutes; c__Negativicutes; o__Veillonellales; f__Veillonellaceae; g__F0422; s__unclassified_mgs_1482
*KANSL3*	2	44	1866	3.35E-07	Saliva	k__Bacteria; p__Patescibacteria; c__Saccharimonadia; o__Saccharimonadales; f__Saccharimonadaceae; g__unclassified_mgs_2626
*EDEM1*	3	86	1987	3.45E-07	Tongue	k__Bacteria; p__Firmicutes; c__Bacilli; o__Lactobacillales; f__Aerococcaceae; g__Granulicatella; s__unclassified_mgs_3116
*SPRTN*	1	86	1996	3.53E-07	Tongue	k__Bacteria; p__Fusobacteriota; c__Fusobacteriia; o__Fusobacteriales; f__Leptotrichiaceae; g__unclassified_mgs_2348
*TBX10*	11	80	1898	3.65E-07	Saliva	k__Bacteria; p__Firmicutes; c__Clostridia; o__TANB77; f__CAG-508; g__CAG-793; s__unclassified_mgs_3234
*EGLN1*	1	225	1996	5.04E-07	Tongue	k__Bacteria; p__Fusobacteriota; c__Fusobacteriia; o__Fusobacteriales; f__Leptotrichiaceae; g__unclassified_mgs_2348
*ATP8A1*	4	554	1904	5.13E-07	Saliva	k__Bacteria; p__Firmicutes; c__Negativicutes; o__Selenomonadales; f__Selenomonadaceae; g__Selenomonas; s__unclassified_mgs_2794
*ZNF277*	7	198	1905	5.27E-07	Saliva	k__Bacteria; p__Bacteroidota; c__Bacteroidia; o__Bacteroidales; f__Bacteroidaceae; g__Prevotella; s__Prevotella_oulorum_mgs_3240
*GJB5*	1	74	1998	5.42E-07	Tongue	k__Bacteria; p__Spirochaetota; c__Spirochaetia; o__Treponematales; f__Treponemataceae; g__Treponema_D; s__unclassified_mgs_1124
*MCHR2*	6	96	1983	7.05E-07	Tongue	k__Bacteria; p__Firmicutes; c__Bacilli; o__Lactobacillales; f__Streptococcaceae; g__Streptococcus; s__unclassified_mgs_449
*LRIF1*	1	49	1894	7.97E-07	Saliva	k__Bacteria; p__Fusobacteriota; c__Fusobacteriia; o__Fusobacteriales; f__Fusobacteriaceae; g__Fusobacterium; s__unclassified_mgs_2180
*EDEM1*	3	86	1976	8.33E-07	Tongue	k__Bacteria; p__Bacteroidota; c__Bacteroidia; o__Bacteroidales; f__Porphyromonadaceae; g__Porphyromonas; s__unclassified_mgs_2117
*ANG*	14	70	1902	9.27E-07	Saliva	k__Bacteria; p__Firmicutes; c__Clostridia; o__Lachnospirales; f__Lachnospiraceae; g__Oribacterium; s__unclassified_mgs_388
*IFNE*	9	52	1894	1.08E-06	Saliva	k__Bacteria; p__Actinobacteriota; c__Actinobacteria; o__Actinomycetales; f__Actinomycetaceae; g__Pauljensenia; s__unclassified_mgs_3546
*RNASE4*	14	81	1902	1.08E-06	Saliva	k__Bacteria; p__Firmicutes; c__Clostridia; o__Lachnospirales; f__Lachnospiraceae; g__Oribacterium; s__unclassified_mgs_388
*EXOC8*	1	27	1996	1.15E-06	Tongue	k__Bacteria; p__Fusobacteriota; c__Fusobacteriia; o__Fusobacteriales; f__Leptotrichiaceae; g__unclassified_mgs_2348
*FZD3*	8	248	1905	1.31E-06	Saliva	k__Bacteria; p__Campylobacterota; c__Campylobacteria; o__Campylobacterales; f__Campylobacteraceae; g__Campylobacter_A; s__Campylobacter_A_showae_A_mgs_1682
*RAI14*	5	540	1995	1.44E-06	Tongue	k__Bacteria; p__Firmicutes; c__Bacilli; o__Erysipelotrichales; f__Erysipelotrichaceae; g__Solobacterium; s__unclassified_mgs_3577
*KCNK9*	8	237	1991	1.45E-06	Tongue	k__Bacteria; p__Firmicutes; c__Bacilli; o__Lactobacillales; f__Streptococcaceae; g__Streptococcus; s__unclassified_mgs_3318
*MUC7*	4	212	1899	2.02E-06	Saliva	k__Bacteria; p__Firmicutes; c__Bacilli; o__Lactobacillales; f__Streptococcaceae; g__Streptococcus; s__unclassified_mgs_1910

**Fig 1:**
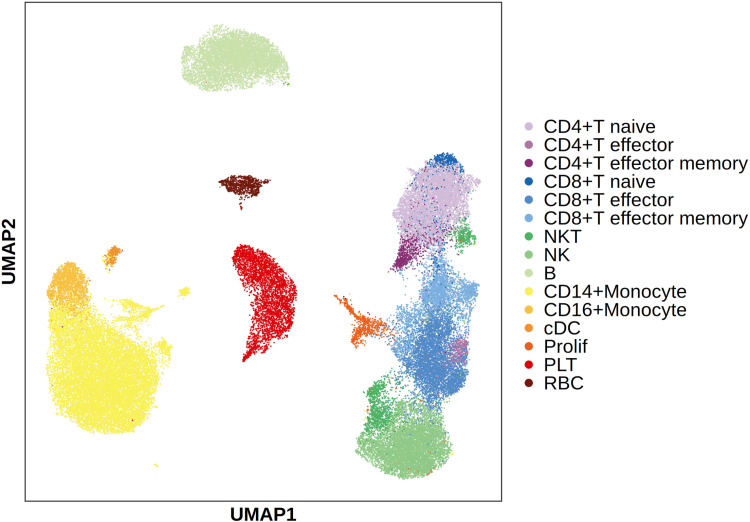
UMAP plot of downscaled scRNA-seq data from severe ILI colored by cell type. Description of data: NK: natural killer cells; cDC: conventional dendritic cells; Prolif: proliferation cells; PLT: platelets; RBC: red blood cells.

For the included ILI-associated single-cell data, a total of 51,243 PBMCs were obtained after QC for subsequent analysis. Downscaling, fractionation and cell type annotation yielded a total of 15 immune cell subtypes. (Fig 1, An additional file shows this in more detail [see S2-S3 Figs]). A total of 4,465 cells from two subtypes, PLTs (n = 3,636) and RBCs (n = 829), were further excluded. A total of 29,149 cells from 13 cell types from severe ILI samples were ultimately included in the flora-associated cell type analysis.

**Fig 2:**
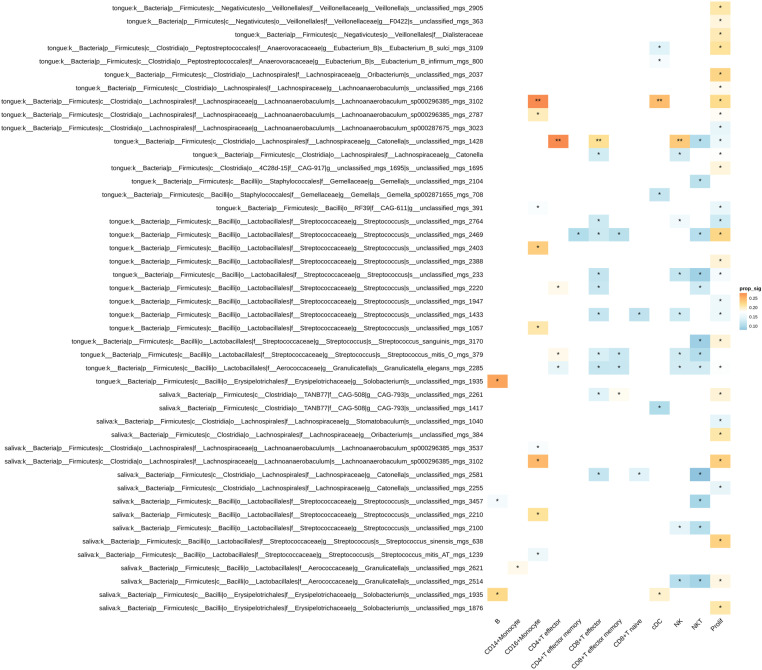
Heatmap of cell type associations with the Firmicutes “phylum” based on scDRS analysis. Description of data: Colors represent the proportion of cells significantly associated with the flora in each cell type, with asterisks indicating significance. *: BH-corrected *P* < 0.05.

According to the results of the scDRS analysis, a total of nine taxa associated with ILI at the “phylum” level were identified. The phylum Firmicutes was the most abundant phylum with 6,305 instances, representing 40.59% of the total, followed by Bacteroidota with 1,846 instances (11.88%), Actinobacteriota with 1,820 instances (11.72%), Proteobacteria with 1,287 instances (8.28%), and Fusobacteriota with 975 instances (6.26%). In total, 205 species were found to be statistically significant, with an adjusted p value (*P*_*adj*_) < 0.05. (**Table 4**).Thirteen immune cell types associated with severe ILI were associated with at least one flora, whereas 205 flora were associated with at least one immune cell type. (An additional file shows this in more detail [see **S4 Fig**]). On the basis of the proportion of significant flora nominally significantly associated cells, CD16 + monocytes were associated with metagenomics-defined Campylobacter_A_concisus_F_mgs_1384 in saliva (29.49%, *P*_*adj*_ = 0.013), unclassified_mgs_803 in the genus Prevotella salivarius (28.9%, *P*_*adj*_ = 0.013), unclassified_mgs_1198 in the genus Haemophilus salivarius (23.43%, *P*_*adj*_ = 0.006), and Lachnoanaerobaculum_sp000296385_mgs_3102 in the genus Lachnoanaerobaculum (27.74%, *P*_*adj*_ = 0.006). The abundance of these flora was significantly correlated with that of other cell types. In this study of the upper respiratory tract flora associated with ILI, Firmicutes was the taxon with the highest proportion at the “phylum” level of categorization, which is consistent with other similar studies [49,50]. Thus, in the study of Firmicutes, 13 immune cell types were associated with at least one flora, and 91 flora were associated with at least one immune cell type.(**Fig 2**) In addition to the significant correlation between Lachnoanaerobaculum_sp000296385_mgs_3102 and the abundance of CD16 + monocytes in the genus Lachnoanaerobaculum described above, this strain was also significantly correlated with the abundance of cDCs (24.48%, *P*_*adj*_ = 0.006). CD4 + T effector cells (27.75%, *P*_*adj*_* *= 0.004), CD8 + T effector cells (21.54%, *P*_*adj*_ = 0.004), and NK cells (23.43%, *P*_*adj*_ = 0.004) were significantly associated with the abundance of unclassified_mgs_1428 in the genus Catonella from the tongue dorsum.

**Table 4:** Based on the “phylum” classification level, the statistical analysis of severe influenza-like illness (ILI) upper respiratory flora.

phylum	Total (%)	Saliva (%)	Tongue (%)	P_sig (%)
Firmicutes	6305(40.59)	2223(35.27)	4082(64.73)	91(1.44)
Bacteroidota	1846(11.88)	975(52.82)	871(47.18)	13(0.70)
Actinobacteriota	1820(11.72)	858(47.14)	962(52.86)	19(1.04)
Proteobacteria	1287(8.28)	507(39.40)	780(60.60)	16(1.24)
Fusobacteriota	975(6.26)	416(42.67)	559(57.33)	10(1.03)
Others	3289(21.17)	1378(41.90)	1911(58.10)	56(1.00)

### 3.2 Identification of cell type-specific genes associated with the upper respiratory tract flora

For the 205 flora identified by scDRS with *P*_*adj*_ < 0.05 and their associated 13 cell types, cell-specific eQTL models were further constructed and analysed by TWAS to identify severe ILI-associated cell type-specific genes associated with the flora.

**Fig 3:**
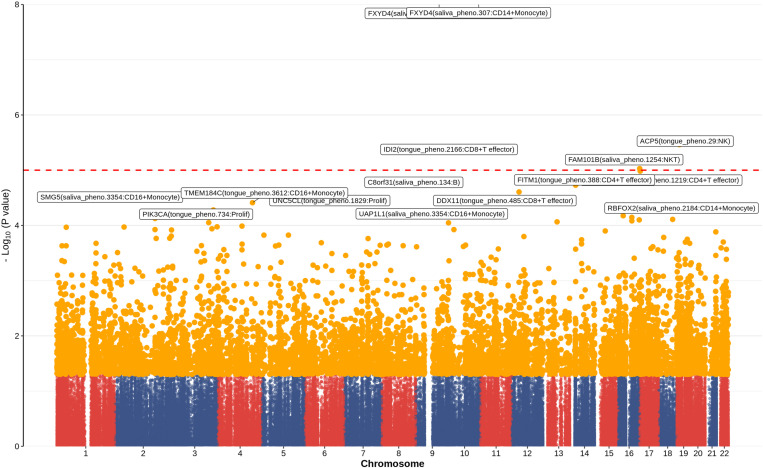
Manhattan plot of cell-specific gene expression and flora associations based on TWAS analysis. Description of data: Red dashed line represents nominal significance level (*P* = 0.05).

A total of 19 significantly associated pairs (*P*_*adj*_ < 0.05) that were closely associated with severe ILI and involved 9 types of immune cells were identified via TWAS analysis. In particular, CD14 + monocyte cells, CD16 + monocyte cells, and CD4 + T effector memory cells had greater numbers of significant associations. Among the 8947 nominally significant association pairs (uncorrected *P* < 0.05), 3854 salivary flora association pairs and 5093 dorsal tongue flora association pairs were identified (Fig 3 and An additional file shows this in more detail [see S1 File]). To further explore their potential roles in ILI, we conducted differential expression analysis on cell type-specific genes associated with particular flora and reported that 1226 genes presented significantly different expression between patients with mild and severe ILI (*P*_*adj*_ < 0.05 and | log_2_FC|>0.25) (An additional file shows this in more detail [see S2 File]). The differential expression analysis results for the 9 cell types significantly correlated with the ILI-associated pairs revealed that *TMEM41B, ERAP2*, and *CCR1* in CD14 + monocyte cells were associated with the metagenomic definition unclassified_mgs_2621 within the genus Granulicatella salivarius (*P*_*adj max*_ < 0.001, log_2_FC_max_ = -0.321). Compared with other immune cells, CD16 + monocyte cells presented the greatest number of DEGs and were significantly associated with 26 types of flora, followed by proliferating cells and cDCs. CD16 + monocyte cells were significantly associated with genes such as *L1TD1*, *MTHFR*, and *ARL6IP5* related to unclassified_mgs_1057 in the genus Streptococcus from the tongue dorsum (*P*_*adj*_ _*max*_ = 0.021, log_2_FC_max_ = -0.257). In CD4 + T effector memory cells, the expression levels of genes such as *CCT3, SEPT2*, and *EFTUD2*, which are associated with Aggregatibacter actinomycetemcomitans_mgs_301 within the genus Aggregatibacter (*P*_*adj max*_ = 0.003, log_2_FCmax = -0.362), were significantly lower in the severe ILI group. Conversely, the expression levels of the gene *SKAP2* associated with Neisseria_sicca_A_mgs_986 in the genus Neisseria from saliva (*P*_*adj max*_ < 0.001, log_2_FC_max_ = 0.473) in CD14 + monocyte cells and the expression levels of genes such as *CFD* and *PSME2* (*P*_*adj max*_ < 0.001, log_2_FC_max_ = 27.591) associated with unclassified_mgs_1057 in the genus Streptococcus from the tongue dorsum and the expression levels of genes such as *TMEM70 and XBP1* (*P*_*adj max*_ < 0.001, log_2_FC_max_ = 3.703) associated with unclassified_mgs_1956 in the genus Prevotella from the tongue dorsum in CD16 + monocyte cells were significantly greater in the severe ILI group.

### 3.3 Functional enrichment analysis of cell type-specific DEGs associated with the flora of the upper respiratory tract

**Fig 4:**
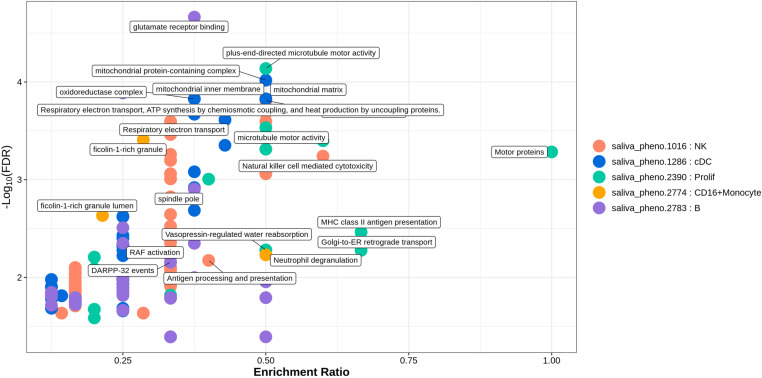
Bubble plot of functional enrichment analysis for salivary flora-associated cell type-specific differentially expressed genes. Description of data: Results of the top 5, arranged in ascending order by q-value; saliva_pheno.1016: Prevotella_loescheii_mgs_2547 within the genus Prevotella in the salivary flora; saliva_pheno.1286: unclassified_mgs_1417 within the genus CAG-793 in the salivary flora; saliva_pheno.2390: unclassified_mgs_2545 within the genus Eikenella in the salivary flora; saliva_pheno.2774: unclassified_mgs_2225 within the genus Campylobacter_A in the salivary flora; saliva_pheno.2783: unclassified_mgs_1397 within the genus Campylobacter_A in the salivary flora.

**Fig 5:**
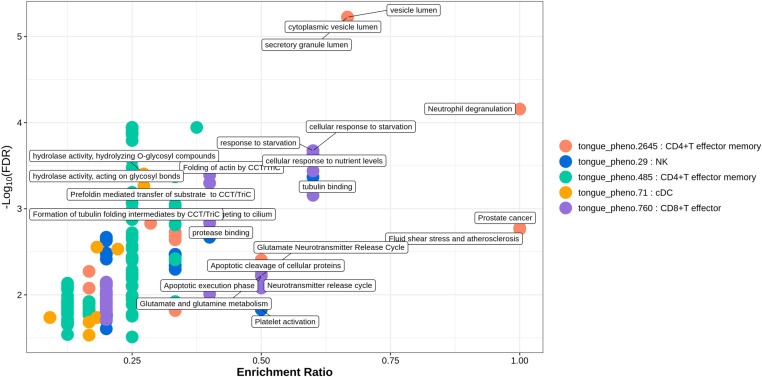
Bubble plot of functional enrichment analysis for dorsal tongue flora-associated cell type-specific differentially expressed genes. Description of data: Results of the top 5, arranged in ascending order by q-value; tongue_pheno.2645: unclassified_mgs_1939 within the genus Campylobacter_A in the dorsal tongue flora; tongue_pheno.29: the genus Catonella in the dorsal tongue flora; tongue_pheno.485: Aggregatibacter_actinomycetemcomitans_mgs_301 within the genus Aggregatibacter in the dorsal tongue flora; tongue_pheno.71: Eubacterium_B_infirmum_mgs_800 within the genus Eubacterium_B in the dorsal tongue flora; tongue_pheno.760: Campylobacter_A_showae_A_mgs_3545 within the genus Campylobacter_A in the dorsal tongue flora.

Functional enrichment analysis based on the GO, KEGG, and Reactome pathway databases was conducted for the identified flora-associated, cell type-specific genes that were significantly differentially expressed between patients with mild and severe ILI. The enrichment results indicated that in CD14 + monocyte cells from ILI patients, differential genes associated with the metagenomic definition unclassified_mgs_2621 (saliva_pheno.2184) in the genus Granulicatella were significantly enriched in processes such as protein carrier chaperone activity, aminopeptidase activity, and intramembrane lipid transporter activity (Enrichment ratio_min_ = 0.167, *FDR*_*max*_ = 0.011), whereas differential genes associated with Neisseria_sicca_A_mgs_986 (saliva_pheno.307) in the genus Neisseria were significantly enriched in biological processes such as the regulation of blood vessel remodelling, negative regulation of extracellular matrix organization, and leucine zipper domain binding (Enrichment ratio_min_ = 0.2, *FDR*_*max*_ = 0.024). The DEGs associated with unclassified_mgs_1939 (tongue_pheno.2645) of the genus Campylobacter_A from the tongue dorsum in CD4 + T effector memory cells were significantly enriched in the processes of the cytoplasmic vesicle lumen, vesicle lumen, and secretory granule lumen (Enrichment ratio_min_ = 0.667, *FDR*_*max*_ < 0.001). Additionally, DEGs associated with Aggregatibacter_actinomycetemcomitans_mgs_301 (tongue_pheno.485) of the genus Aggregatibacter from the tongue dorsum were significantly enriched in the biological processes of the regulation of establishment of protein localization to telomeres, the regulation of protein localization to the Cajal body, and the positive regulation of protein localization to the Cajal body (Enrichment ratio_min_ = 0.250, *FDR*_max_ < 0.001). Differential genes in cDC associated with unclassified_mgs_1417 (saliva_pheno.1286) from the genus saliva CAG-793 were enriched in processes such as mitochondrial protein-containing complexes, oxidoreductase complexes, and the mitochondrial matrix (Enrichment ratio_min_ = 0.375, *FDR*_max_ < 0.001). Additionally, differential genes associated with Eubacterium_B_infirmum_mgs_800 (tongue_pheno.71) from the dorsal tongue Eubacterium were significantly enriched in biological processes related to hydrolase activity, hydrolysis of O-glycosyl compounds, and other pathways involving the degradation of glycosyl bonds and sugars. In contrast, NK cells were significantly enriched for biological processes associated with differential gene enrichment, with both saliva Prevotella_loescheii_mgs_2547 (saliva_pheno.1016) and tongue dorsum Catonella (tongue_pheno.29), and Prevotella_loescheii_mgs_2547 mgs_2547 were enriched for biological processes such as regulation of leukocyte-mediated cytotoxicity and regulation of cell killing (Enrichment ratio_min_ = 0.500, *FDR*_max_ = 0.007). The genus Catonella was enriched for tubulin binding, Polycomb repressive complex and other processes (Enrichment ratio_min_ = 0.400, *FDR*_max_ = 0.015). The unclassified_mgs_1397 (saliva_pheno.2783)-related DEGs in the genus Saliva Campylobacter_A were enriched mainly in biological processes such as glutamate receptor binding, spindle pole and dopaminergic synapse; pathways related to neurodegeneration; multiple diseases; and other biological pathways (Enrichment ratio_min_ = 0.333, *FDR*_max_ = 0.041). Differential genes associated with saliva Eikenella 2545 unclassified_mgs_2545 (saliva_pheno.2390) were enriched in proliferating cells for motor proteins, microtubule motor activity, vasopressin-regulated water reabsorption and other biological processes (Enrichment ratio_min_ = 0.500, *FDR*_max_ = 0.005). Differential genes associated with saliva unclassified_mgs_2225 (saliva_pheno.2774) and tongue dorsal Campylobacter_A_showae_A_mgs_3545 (tongue_pheno.760) in the genus Campylobacter_A were associated with CD16 + monocyte cells and CD8 + T effector cells enriched in the following biological processes: ficolin-1-rich granules, ficolin-1-rich granule lumen (Enrichment ratio_min_ = 0. 214, *FDR*_max_ = 0.002), the response to starvation, the cellular response to nutrient levels, and the cellular response to starvation (Enrichment ratio_min_ = 0.600, *FDR*_max_ < 0.001).(Figs 4 and 5, Table 5, and An additional file shows this in more detail [see S3 File]).

**Table 5:** The functional enrichment analysis of cell type-specific expression genes related to flora community.

Bacterial community	Cell Type	Data Base	Pathway/Biological Process	Enrichment Ratio	*FDR*
			aminopeptidase activity	0.167	0.010
			intramembrane lipid transporter activity	0.167	0.010
			calcium-dependent phospholipid binding	0.167	0.011
			chemokine binding	0.167	0.008
saliva:k__Bacteria; p__Proteobacteria; c__Gammaproteobacteria; o__Burkholderiales; f__Neisseriaceae; g__Neisseria; s__Neisseria_sicca_A_mgs_986	CD14 + Monocyte	GO	regulation of blood vessel remodeling	0.2	0.023
			negative regulation of extracellular matrix organization	0.2	0.023
			negative regulation of collagen metabolic process	0.2	0.023
			regulation of extracellular matrix disassembly	0.2	0.023
			cotranslational protein targeting to membrane	0.2	0.024
			glycoprotein catabolic process	0.2	0.024
			leucine zipper domain binding	0.2	0.011
			LRR domain binding	0.2	0.011
**Bacterial community**	**Cell Type**	**Data Base**	**Pathway/Biological Process**	**Enrichment Ratio**	***FDR***
tongue:k__Bacteria; p__Campylobacterota; c__Campylobacteria; o__Campylobacterales; f__Campylobacteraceae; g__Campylobacter_A; s__unclassified_mgs_1939	CD4 + T effector memory	GO	secretory granule lumen	0.7	5.95E-06
			cytoplasmic vesicle lumen	0.7	5.95E-06
			vesicle lumen	0.7	5.95E-06
		KEGG	Prostate cancer	1	0.002
			Fluid shear stress and atherosclerosis	1	0.002
saliva:k__Bacteria;p__Campylobacterota; c__Campylobacteria; o__Campylobacterales; f__Campylobacteraceae; g__Campylobacter_A; s__unclassified_mgs_1397	B	GO	glutamate receptor binding	0.375	2.17E-05
			spindle pole	0.375	0.001
		KEGG	Pathways of neurodegeneration - multiple diseases	0.5	0.041
			Dopaminergic synapse	0.333	0.041
			Oocyte meiosis	0.333	0.041
			Fluid shear stress and atherosclerosis	0.333	0.041
			Adrenergic signaling in cardiomyocytes	0.333	0.041
			Cellular senescence	0.333	0.041
			Autophagy - animal	0.333	0.041
**Bacterial community**	**Cell Type**	**Data Base**	**Pathway/Biological Process**	**Enrichment Ratio**	***FDR***
saliva:k__Bacteria; p__Proteobacteria; c__Gammaproteobacteria; o__Burkholderiales; f__Neisseriaceae; g__Eikenella; s__unclassified_mgs_2545	Proliferating cell	GO	plus-end-directed microtubule motor activity	0.5	7.29E-05
			microtubule motor activity	0.5	< 0.001
		KEGG	Motor proteins	1	0.001
			Vasopressin-regulated water reabsorption	0.5	0.001
saliva:k__Bacteria; p__Firmicutes; c__Clostridia; o__TANB77; f__CAG-508; g__CAG-793; s__unclassified_mgs_1417	cDC	GO	mitochondrial protein-containing complex	0.5	9.60E-05
			oxidoreductase complex	0.375	< 0.001
			mitochondrial matrix	0.5	< 0.001
			mitochondrial inner membrane	0.5	< 0.001
			inner mitochondrial membrane protein complex	0.375	< 0.001
**Bacterial community**	**Cell Type**	**Data Base**	**Pathway/Biological Process**	**Enrichment Ratio**	***FDR***
tongue:k__Bacteria; p__Proteobacteria; c__Gammaproteobacteria; o__Enterobacterales; f__Pasteurellaceae; g__Aggregatibacter; s__Aggregatibacter_actinomycetemcomitans_mgs_301	CD4 + T effector memory	GO	positive regulation of establishment of protein localization to telomere	0.25	< 0.001
			regulation of establishment of protein localization to telomere	0.25	< 0.001
			regulation of protein localization to Cajal body	0.25	< 0.001
			positive regulation of protein localization to Cajal body	0.25	< 0.001
			regulation of establishment of protein localization to chromosome	0.25	< 0.001
			protein localization to nuclear body	0.25	< 0.001
			positive regulation of protein localization to chromosome, telomeric region	0.25	< 0.001
			protein localization to Cajal body	0.25	< 0.001
saliva:k__Bacteria;p__Bacteroidota; c__Bacteroidia; o__Bacteroidales; f__Bacteroidaceae; g__Prevotella; s__Prevotella_loescheii_mgs_2547	NK	GO	regulation of leukocyte mediated cytotoxicity	0.5	< 0.001
			regulation of cell killing	0.5	< 0.001
		KEGG	Natural killer cell mediated cytotoxicity	0.6	0.001
			Antigen processing and presentation	0.4	0.007
**Bacterial community**	**Cell Type**	**Data Base**	**Pathway/Biological Process**	**Enrichment Ratio**	***FDR***
tongue:k__Bacteria; p__Campylobacterota; c__Campylobacteria; o__Campylobacterales; f__Campylobacteraceae; g__Campylobacter_A; s__Campylobacter_A_showae_A_mgs_3545	CD8 + T effector	GO	cellular response to starvation	0.6	< 0.001
			response to starvation	0.6	< 0.001
			cellular response to nutrient levels	0.6	< 0.001
saliva:k__Bacteria; p__Campylobacterota; c__Campylobacteria; o__Campylobacterales; f__Campylobacteraceae; g__Campylobacter_A; s__unclassified_mgs_2225	CD16 + Monocyte	GO	ficolin-1-rich granule	0.288	< 0.001
			ficolin-1-rich granule lumen	0.214	0.002
tongue:k__Bacteria;; p__Firmicutes; c__Clostridia; o__Peptostreptococcales; f__Anaerovoracaceae; g__Eubacterium_B; s__Eubacterium_B_infirmum_mgs_800	cDC	GO	hydrolase activity, hydrolyzing O-glycosyl compounds	0.273	< 0.001
			hydrolase activity, acting on glycosyl bonds	0.273	0.001
		KEGG	Other glycan degradation	0.222	0.003
**Bacterial community**	**Cell Type**	**Data Base**	**Pathway/Biological Process**	**Enrichment Ratio**	***FDR***
tongue:k__Bacteria; p__Firmicutes; c__Clostridia; o__Lachnospirales; f__Lachnospiraceae; g__Catonella	NK	GO	tubulin binding	0.6	< 0.001
			protease binding	0.4	0.001
		KEGG	Polycomb repressive complex	0.5	0.015
			Platelet activation	0.5	0.015

saliva_pheno.2184: The stran identified as unclassified_mgs_2621 within the genus Granulicatella, isolated from the saliva, was defined through metagenomic analysis; saliva_pheno.307: The stran identified as unclassified_mgs_986 within the genus Neisseria, isolated from the saliva, was defined through metagenomic analysis; tongue_pheno.2645:The stran identified as unclassified_mgs_1939 within the genus Campylobacter_A, isolated from the dorsal surface of the tongue, was defined through metagenomic analysis; saliva_pheno.2783:The stran identified as unclassified_mgs_1397 within the genus Campylobacter_A, isolated from the saliva, was defined through metagenomic analysis; saliva_pheno.2390:The stran identified as unclassified_mgs_2545 within the genus Eikenella, isolated from the saliva, was defined through metagenomic analysis; saliva_pheno.1286:The strain unclassified_mgs_1417, defined by 16S rRNA gene sequencing, within the genus associated with saliva CAG-793; tongue_pheno.485:Within the genus Aggregatibacter, Aggregatibacter_actinomycetemcomitans_mgs_301 is a rod-shaped stran isolated from the dorsal surface of the tongue; saliva_pheno.1016: Within the genus Prevotella, Prevotella_loescheii_mgs_2547 is a strain isolated from saliva; tongue_pheno.760:Within the genus Campylobacter_A, Campylobacter_A_showae_A_mgs_3545 is a strain isolated from the dorsal surface of the tongue;saliva_pheno.2774:The stran identified as unclassified_mgs_2225 within the genus Campylobacter_A, isolated from the saliva, was defined through metagenomic analysis; tongue_pheno.71:Within the genus Eubacterium_B, Eubacterium_B_infirmum_mgs_800 is a strain isolated from the dorsal surface of the tongue; tongue_pheno.29: Within the genus Catonella, isolated from the dorsal surface of the tongue.

## 4. Discussion

This study integrated upper respiratory tract flora-related GWAS data and severe ILI-related transcriptomic data to explore the potential associations between upper respiratory tract flora and immune cells in severe ILI and to investigate the possible mechanisms influencing severe ILI from the perspectives of metabolism and the immune response.

The study employed a stratified design to integrate multi-source heterogeneous data: the upper respiratory tract flora GWAS data were derived from a homogeneous cohort of healthy Han Chinese individuals, including 2017 tongue dorsum samples and 1915 saliva samples, while transcriptomic data related to severe ILI were integrated from independent cohorts of Southeast Asian and European populations. Previous studies have shown that host genetic factors exhibit long-term stability in the relative abundance of heritable flora taxa, with minimal disruption by environmental factors [51]. Therefore, this study effectively reduced the interference of host heterogeneity and environmental confounding factors in the flora-immune association analysis by including a cohort with consistent genetic background, strict health status screening, and standardized sampling sites. Further research has shown that the diversity of human immune genes is driven by human contact with pathogens [52,53], and there is a significant correlation between genetic variability and global pathogen diversity [54]. Some of these correlations may be confounded by variables such as climate, diet, or lifestyle. Thus, incorporating samples with diverse genetic backgrounds can reveal the heterogeneity of immune responses across different populations.

The primary objective of this study is to investigate the common immunological mechanisms underlying influenza-like illness (ILI) and their associations with the upper respiratory flora and host immune response, rather than to elucidate pathogen-specific mechanisms. Moreover, the early symptoms of COVID-19 substantially overlap with those of ILI, and in severe cases, both conditions manifest with upper respiratory inflammation and immune dysregulation. Therefore, we regard COVID-19, in essence, as a form of ILI, and it is classified as part of ILI in public health surveillance. To encompass a broader range of ILI and a wider spectrum of ILI samples, we included patients with ILI caused by various pathogens. This design not only reveals the common features of upper respiratory immune responses and alterations in the flora but also represents the broad clinical phenotype of ILI.

Through flora-associated cell type analysis, we identified a total of 205 flora associated with 13 immune cell subtypes, with each immune cell subtype identifying 1–4 flora. Notably, statistically significant associations were identified between 205 species of flora and at least one immune cell types, which may reveal the potential regulatory roles of flora in modulating immune responses.In the gastrointestinal tract, the characteristic feature of acute mucosal infections is dysbiosis, accompanied by significant changes in flora and bacterial dominance. This dysbiotic state may enhance pathogen invasiveness and local inflammatory responses, thereby exacerbating inflammation and tissue damage [55].Previous studies have shown that interactions within the flora, mediated by metabolites that modulate innate immune responses, regulate synergistic mechanisms that drive the stability of flora. Therefore, the correlation between multiple flora and at least one immune cell type may help explain why the upper respiratory tract flora does not rely on a single or few “key flora,” but rather exerts its effects through a complex regulatory network formed by the overall composition of the community, thereby reflecting the collective function of the flora [56].The surface components of the Granulicatella flora can activate CD14 + monocyte cells via the Toll-like receptor, which recognizes pathogens through pathogen-associated molecular patterns. ligation of Toll-like receptor - pathogen-associated molecular patterns initiates Toll-like receptor signalling, which is critical for the innate immune cascade that induces cytokine or chemokine secretion and cell recruitment for pathogen clearance [57]. In addition, these CD14 + monocytes eliminate bacteria via phagocytosis and initiate further immune responses by differentiating into macrophages or dendritic cells [57]. This flora influences the phenotype of ILI by modulating the response of the body’s immune cells. Another study revealed that NK cells play a critical role in antiviral and antitumour immunity. The function of NK cells is regulated by their surface CD16 receptors, a process that can be influenced by different bacterial populations. In HIV-1-infected individuals, the number of CD16 + cells increases [58], and the interaction between Campylobacterota flora and NK cells may influence the host immune response by modulating NK cell activity and quantity. This study revealed that these associations may be partially related to their roles in phagocytosis and antiviral activities in CD14 + monocyte cells, NK cells, and CD16 + monocyte cells, as well as in regulating NK cell activity. Campylobacter_A_showae_A_mgs_3545 from the genus Campylobacter_A on the dorsal tongue and unclassified_mgs_2621 from the genus Granulicatella in saliva Infection with the two flora can result in local and systemic immune responses, including the activation and differentiation of CD4 + naive T cells, which, upon encountering Campylobacter_A_showae antigens displayed by antigen-presenting cells, further differentiate into helper T cells (Th1, Th2, Th17) and regulatory T cells that regulate the immune response. As previous studies have shown, the genus Streptococcus is a common flora in the oral cavity and stimulates naive CD8 + T cells through antigen-presenting cells, leading to their activation and differentiation into cytotoxic T lymphocytes, which, in the local environment of the oral cavity, can directly kill infected epithelial cells and control the spread of bacterial infection and coinfection with viruses and can directly kill infected epithelial cells and control the spread of bacterial infection and coinfection with viruses, thereby reducing the bacterial load, which peaks after 7 days of ILI infection [59,60]. In this setting, other associated immune cells show reduced phagocytosis and increased levels of oxidative free radicals, associated with the suppression of proinflammatory cytokine secretion. In the present study, the Streptococcus genus flora was hypothesized to effectively activate CD8 + naive T cells via an antigen presentation mechanism, prompting them to differentiate into highly efficient CD8 + T cells, which actively participate in the viral clearance process. The extent of ILI infection was significantly controlled, effectively preventing its progression to more severe stages. Furthermore, these CD8 + T cells further differentiate into CD8 + T effector memory cells, which may provide long-lasting protection for future immune defense, enhancing the body’s ability to fight against similar pathogens. Studies have shown that the Firmicutes phylum not only plays a critical role in maintaining intestinal homeostasis but also affects CD16 + monocyte function through its metabolites. These metabolites can enhance the anti-inflammatory and immunomodulatory functions of monocytes by regulating their phenotype, thereby providing protection against infections and chronic inflammatory diseases [61]. Among all significantly identified flora in this study, seven phyla were found to be correlated with NK cells, CD16 + monocytes, CD14 + monocyte cells, and the metabolites of the flora may affect the regulation of the phenotype of mononuclear immune cells and their anti-inflammatory and immunomodulatory functions. In patients with severe ILI, changes in the abundance of the Firmicutes phylum in the blood may affect the expression of contact-dependent genes in CD16 + monocyte cells by regulating the expression of inflammation-related proteins such as CX3CR1-CX3CL1 and IL-1β, thereby influencing the body’s immune recovery capacity [62]. The abundance of the Firmicutes and Campylobacterota phyla was particularly notable

In addition, certain strains of the Prevotella genus have complex relationships with human health, with some strains being associated with inflammation, metabolic diseases, and immune responses [63]. Prevotella flora stimulate epithelial cells to produce IL-8, IL-6, and CCL20, thereby promoting mucosal Th17 immune responses and neutrophil recruitment. Prevotella genus-mediated mucosal inflammation leads to the systemic dissemination of inflammatory mediators, bacteria, and bacterial products, which in turn may influence systemic disease outcomes [64]. The associations found in the present study between these strains and various responses and inflammatory factors may be related to their effects on the activity of specific B cells, CD4 + T effector cells, and NK cells, as well as the expression of specific genes or proteins. Further research is needed to confirm these findings.

In the present study, certain genes associated with the genera Streptococcus CD14 + monocyte cells, NK cells and CD16 + monocyte cells also exhibited significant differential expression between the severe and mild ILI groups. Functional enrichment analysis of these DEGs revealed that in the genus Streptococcus, the DEGs associated with the dorsum of the tongue metagenome, unclassified_mgs_1057, were significantly enriched in pathways related to nuclear androgen receptor binding and transcription coactivator activity. These findings suggest that the genus Streptococcus tongue dorsalis may influence the progression of ILI by initiating the transcription of specific genes in the nucleus of CD16 + monocyte cells that interact with the cellular regulatory RNA polymerase II complex. Similarly, certain genes associated with Eubacterium in cDCs were similarly and significantly differentially expressed between the severe and mild ILI groups. Further functional enrichment analysis of these differentially expressed genes revealed that those associated with Eubacterium B infirmum mgs 800 on the dorsum of the tongue were significantly enriched in processes related to the azurophil granule membrane, lysosomal lumen, hydrolase activity, glycosidase activity, and transcription initiation factor binding. This finding suggests a mechanism by which the strain may influence the course of ILI disease by regulating enzyme activity, activating transcription factors, and regulating gene expression in combination with specific DNA sequences. The present study also revealed that the dorsal tongue unclassified_mgs_1939 of the genus Campylobacter_A was associated with CD4 + T effector memory in patients with mild ILI with differential gene expression. TWAS and pathway enrichment analysis revealed that these DEGs were enriched in biological processes such as the secretory granule lumen, prostate cancer, fluid shear stress and atherosclerosis. These findings suggest that the effect of the dorsal tongue metagenomics definition unclassified_mgs_1939 on the body’s infection-associated immune response may be related to its effect on regulatory signalling in CD4 + T effector memory cells or the production of immunosuppressive molecules in CD4 + T effector memory cells. However, “enrichment” does not necessarily imply that these genes exhibit higher expression levels in severe patients; rather, it reflects a stronger association or activation trend of these genes within specific immune responses or signaling pathways. Regarding the differential regulation of immune cell gene expression by the same associated flora, the upper respiratory tract flora can directly regulate immune cell gene expression through its metabolites, thereby leading to differences in gene expression within the same immune cell type. Severe ILI patients typically exhibit a more pronounced immune response, this immune state is closely associated with the interaction between the host and the upper respiratory tract flora. The same flora may exert distinct biological effects under different immune conditions, ultimately contributing to differences in immune cell gene expression.

The main limitations of the present study are as follows. First, the integrative analysis for identifying flora-associated cell types was based on the assumption that interindividual differences in the abundance of upper respiratory tract flora could be attributed to genetic variation to some extent. Although the study acquired all the data of upper respiratory tract flora-associated genome-wide association studies from the National Gene Bank of China database and only the GWAS summary data with h2 > 0.1 were included in the integration analysis, the GWAS revealed only the association between genetic variation and phenotype rather than direct causality, while the mechanism of the effect of fewer heritable flora on ILI remained unclear. Second, the relatively small sample size (n = 2984) of the GWAS pooled data included in this study may be one of the reasons for the unsatisfactory signals of the integrated analysis and the TWAS analysis. In addition, in the identification of flora-associated cell type-specific expressed genes, the eQTL model used was constructed from the EUR population, and although genomic data from different races were used as reference panels in the model construction (EUR) and TWAS analysis (EAS), differences in genetic structure between races may still be an important reason why fewer significant genes were identified in this part of the study than in similar studies. Finally, this part of the study investigated only the associations between flora and immune cells from a transcriptional and metabolic perspective, and additional epigenetic and protein studies may be considered to gain a more comprehensive understanding of the underlying mechanisms involved in “upper respiratory flora-immunity-ILI progression”. Moreover, the conclusions of the bioinformatics analysis should be further verified by experimental and population studies.

In summary, a total of 205 flora associated with 13 cell types were analysed in this study, and cell-specific eQTL models were further constructed and analysed via TWAS. On the basis of this analysis, 1226 flora-associated cell type-specific genes with significantly different expression levels between patients with mild and severe ILI were identified (*P*_*adj*_ < 0.05 and | log_2_FC|>0.25). Finally, by analysing the genes specifically expressed in cell types associated with the significantly differentially expressed flora, pathways or biological processes related to ILI progression were enriched.

## Supporting information

S1 Supporting information This zip file contains supplementary files, figures to the study.
(ZIP)

## Abbreviations

ILI: influenza like illness
Treg: regulatory T cells
COVID-19: novel coronavirus pneumonia
QC: quality control
KO: Kyoto Encyclopedia of Genes and Genomes
BH: Benjamini & Hochberg
FDR: false discovery rate
COPD: chronic obstructive pulmonary disease
GWAS: genome-wide association studies
CNGBdb: China National Gene Bank database
WGS: whole genome sequencing
SNPs: single nucleotide polymorphisms
MAF: minor allele frequency
EAS: East Asian
EUR: European
scRNA-seq: single-cell RNA sequencing
GEO: Gene Expression Omnibus
PBMC: peripheral blood mononuclear cell
UMAP: uniform manifold approximation and projection
PLT: platelet
RBC: red blood cell
MC: Monte Carlo
LD: linkage disequilibrium
eQTL: expression quantitative trait loci
TWAS: transcriptome-wide association analysis
GO: Gene Ontology
FC: fold change
NK: natural killer cells
cDC: conventional dendritic cells
Prolif: proliferation cells
