# Supplementary figures and images for: Leveraging transcriptome-wide association studies identifies the relationship between upper respiratory flora and cell type-specific gene expression in severe respiratory disease
Source: PLoS One. 2025 May 9;20(5):e0322864. doi: 10.1371/journal.pone.0322864 (PMC12063895; doi:10.1371/journal.pone.0322864)

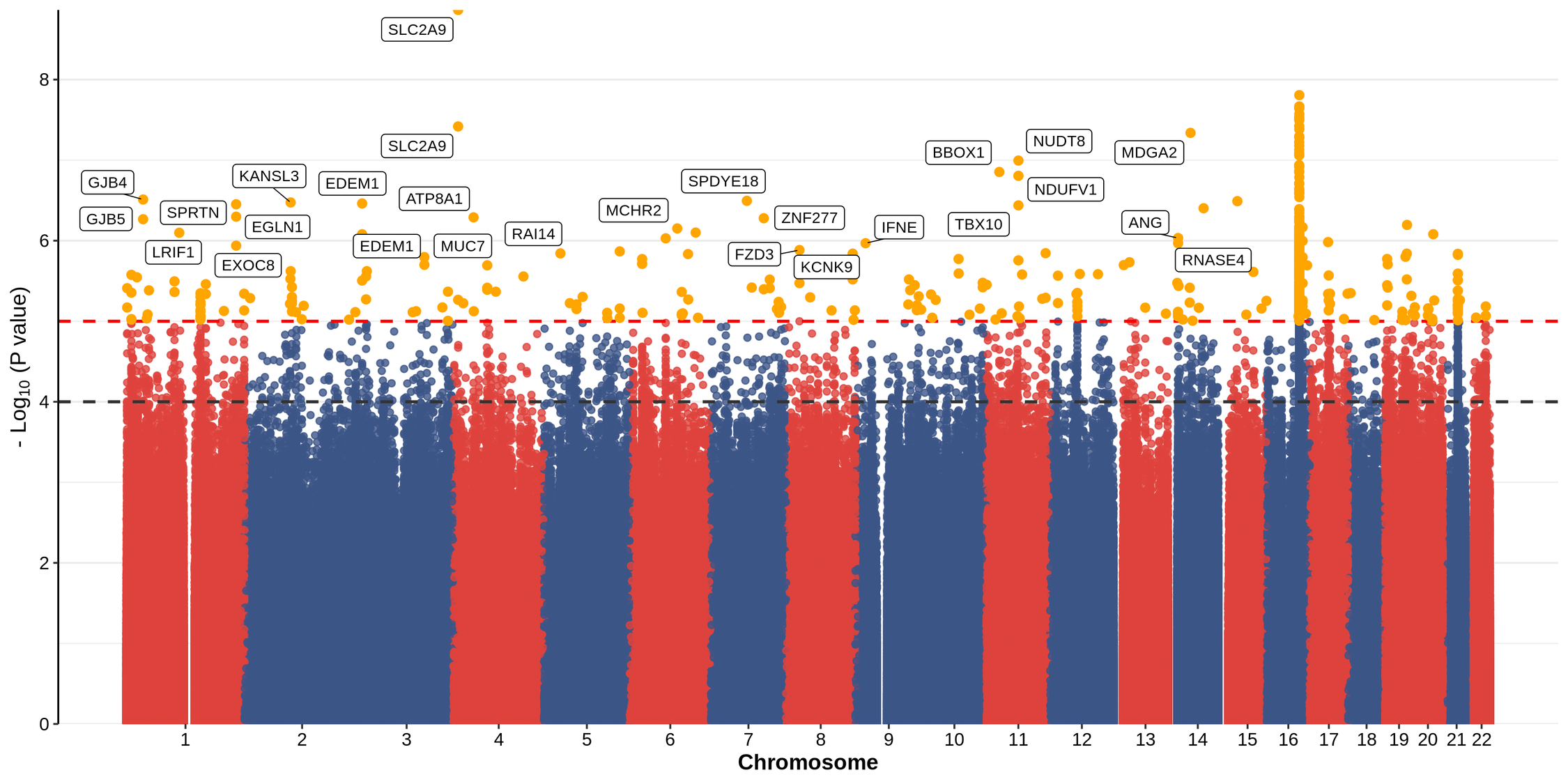

Supplement: S1 Supporting information — This zip file contains supplementary files, figures to the study. (ZIP) [file pone.0322864.s001.zip › S1 Fig.tif]

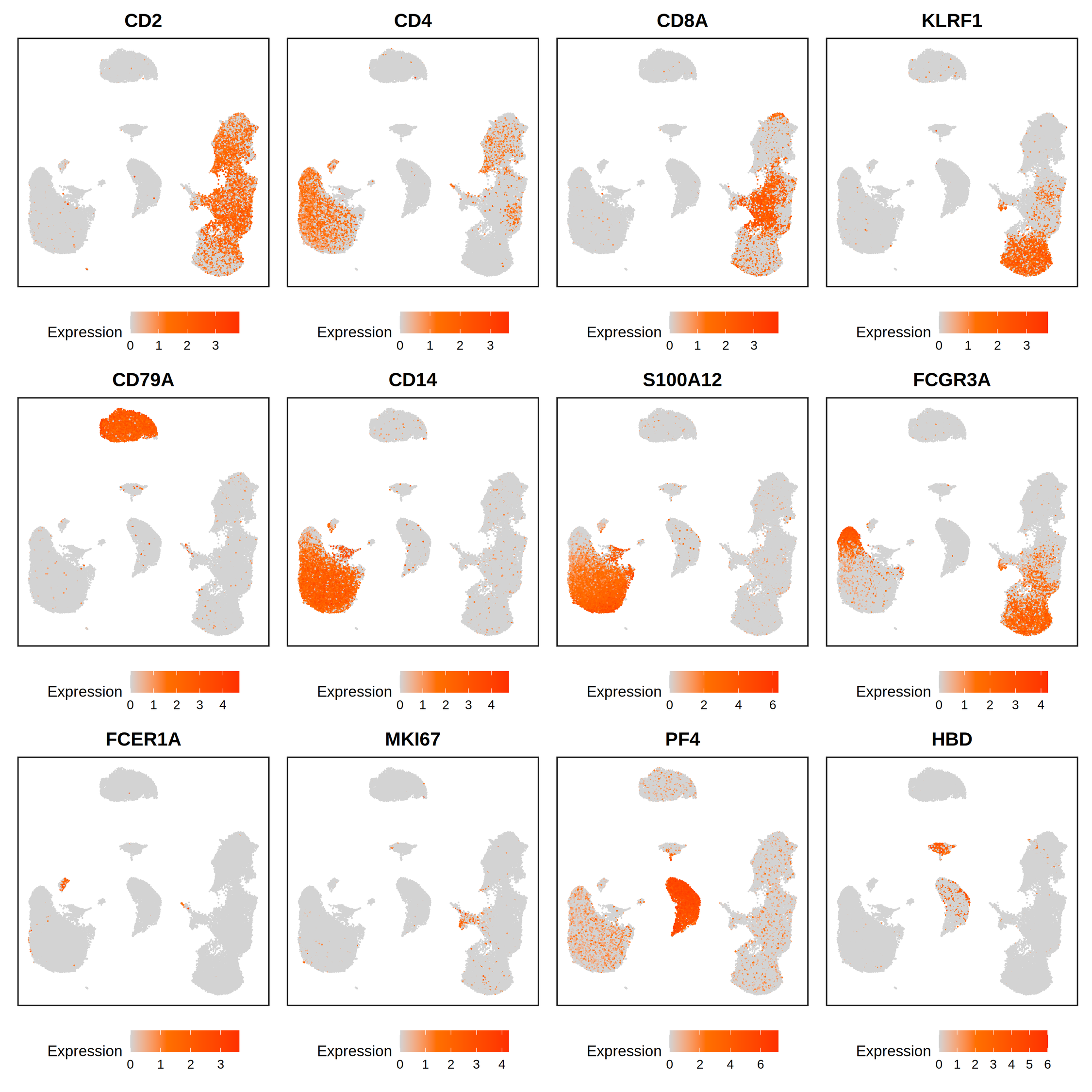

Supplement: S1 Supporting information — This zip file contains supplementary files, figures to the study. (ZIP) [file pone.0322864.s001.zip › S2 Fig.tif]

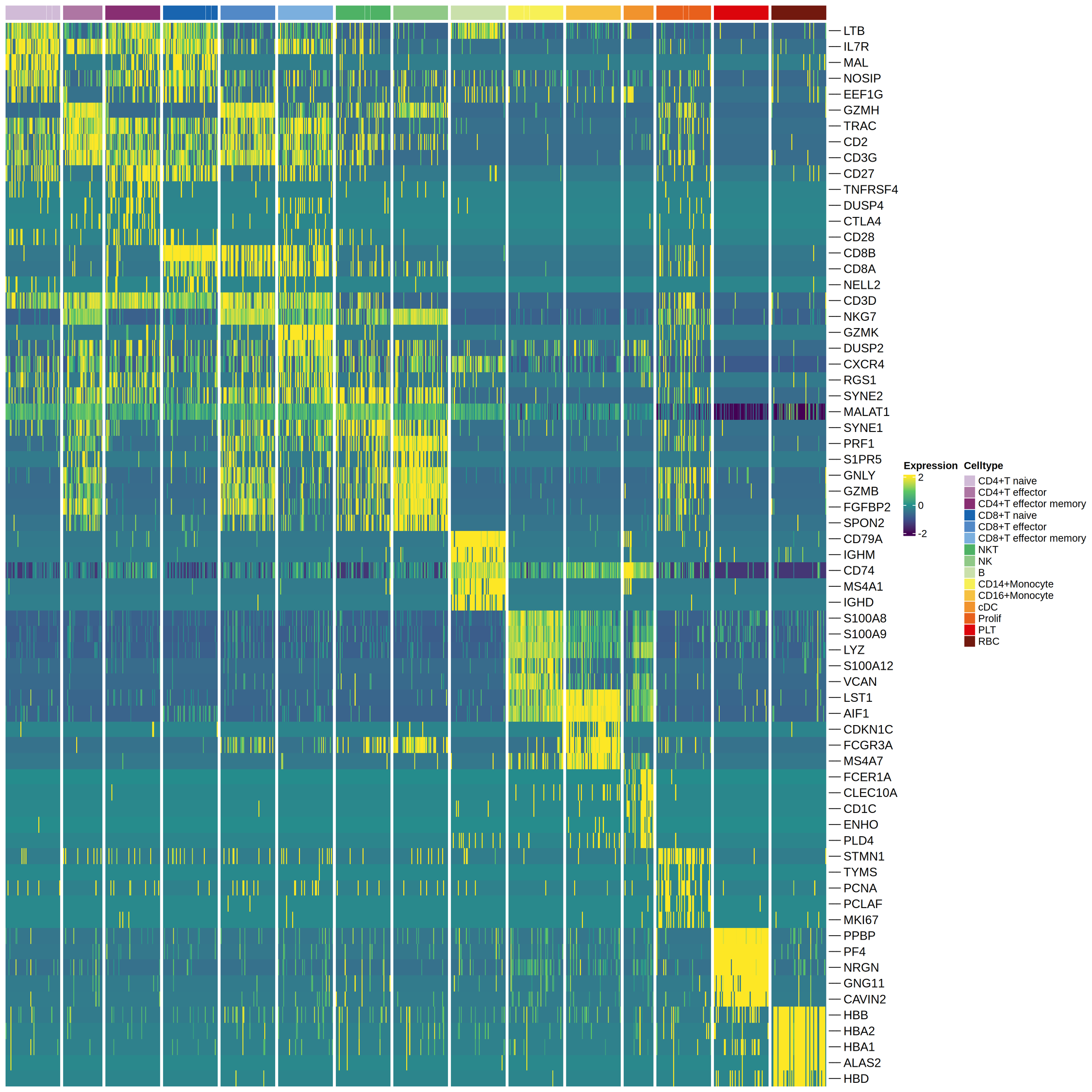

Supplement: S1 Supporting information — This zip file contains supplementary files, figures to the study. (ZIP) [file pone.0322864.s001.zip › S3 Fig.tif]

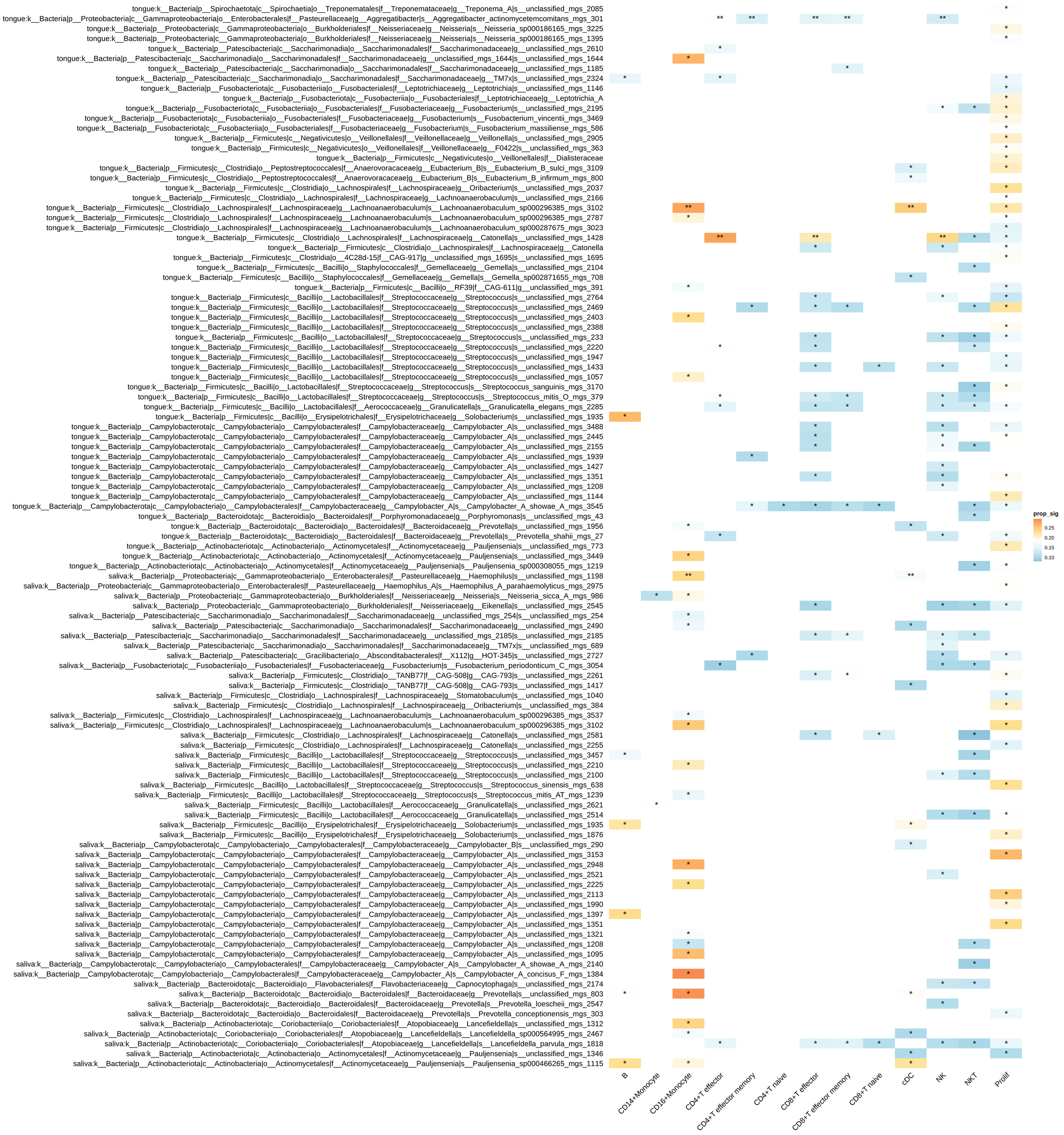

Supplement: S1 Supporting information — This zip file contains supplementary files, figures to the study. (ZIP) [file pone.0322864.s001.zip › S4 Fig.tif]
